# ADHD in girls and boys – gender differences in co-existing symptoms and executive function measures

**DOI:** 10.1186/1471-244X-13-298

**Published:** 2013-11-09

**Authors:** Erik Winther Skogli, Martin H Teicher, Per Normann Andersen, Kjell Tore Hovik, Merete Øie

**Affiliations:** 1 Innlandet Hospital Trust Lillehammer, Division Mental Health Care, Oslo, Norway; 2 Harvard Medical School, Boston, USA; 3 Department of Psychology, University of Oslo, Innlandet Hospital Trust Lillehammer, Division Mental Health Care, Oslo, Norway

**Keywords:** ADHD, Gender, Comorbidity, Executive functions, BRIEF

## Abstract

**Background:**

ADHD is diagnosed and treated more often in males than in females. Research on gender differences suggests that girls may be consistently underidentified and underdiagnosed because of differences in the expression of the disorder among boys and girls. One aim of the present study was to assess in a clinical sample of medication naïve boys and girls with ADHD, whether there were significant gender x diagnosis interactions in co-existing symptom severity and executive function (EF) impairment. The second aim was to delineate specific symptom ratings and measures of EF that were most important in distinguishing ADHD from healthy controls (HC) of the same gender.

**Methods:**

Thirty-seven females with ADHD, 43 males with ADHD, 18 HC females and 32 HC males between 8 and 17 years were included. Co-existing symptoms were assessed with self-report scales and parent ratings. EF was assessed with parent ratings of executive skills in everyday situations (BRIEF), and neuropsychological tests. The three measurement domains (co-existing symptoms, BRIEF, neuropsychological EF tests) were investigated using analysis of variance (ANOVA) and random forest classification.

**Results:**

ANOVAs revealed only one significant diagnosis x gender interaction, with higher rates of self-reported anxiety symptoms in females with ADHD. Random forest classification indicated that co-existing symptom ratings was substantially better in distinguishing subjects with ADHD from HC in females (93% accuracy) than in males (86% accuracy). The most important distinguishing variable was self-reported anxiety in females, and parent ratings of rule breaking in males. Parent ratings of EF skills were better in distinguishing subjects with ADHD from HC in males (96% accuracy) than in females (92% accuracy). Neuropsychological EF tests had only a modest ability to categorize subjects as ADHD or HC in males (73% accuracy) and females (79% accuracy).

**Conclusions:**

Our findings emphasize the combination of self-report and parent rating scales for the identification of different comorbid symptom expression in boys and girls already diagnosed with ADHD. Self-report scales may increase awareness of internalizing problems particularly salient in females with ADHD.

## Background

Attention-deficit/hyperactivity disorder (ADHD) is one of the most common childhood neuropsychiatric disorders, characterized by problems with inattention, hyperactivity and impulsivity [1]. Worldwide prevalence estimates for childhood ADHD range between 3% and 7% [1] with a male-to-female ratio of 3:1 in population based studies [2,3] and between 5:1 to 9:1 in clinical samples [1,3,4]. Research on gender differences suggests that girls may be consistently underidentified and underdiagnosed mostly explained by differences in the expression of the disorder among boys and girls [3,5-7]. Females with ADHD are reported to have fewer hyperactive/impulsive symptoms and more inattentive symptoms when compared with males with ADHD [3,8,9]. Further, females with ADHD present more commonly with the inattentive subtype than do boys [10]. Less disruptive behavior in females with ADHD may contribute to referral bias causing underidentification and lack of treatment for females with ADHD [11]. For example, Sciutto, Nolfi, & Bluhm [12] found that teachers more often refer boys than girls for treatment for ADHD, even when showing equal levels of impairment.

Another major contributing factor to late or missed diagnoses in females appears to be the presence of co-existing symptoms that often cloud the diagnostic picture [5]. As many as 75% of children with ADHD are likely to have at least one other psychiatric disorder [2,13]. Thus, in clinical practice, co-existing psychiatric problems are the rule rather than the exception. Boys with ADHD have been found to have more externalizing disorders than normal developing boys, while females tend to show more internalizing disorders in comparison to normal developing girls [3,5,9]. In addition, adolescent females with ADHD are reported to show more internalizing symptoms than their male counterparts [14]. Often other diagnoses are made long before the diagnosis of ADHD is assessed in female clinical populations because of less overt ADHD symptoms [15]. By comparison, more overt acting out behavior seems to drive referral for ADHD assessment in boys [3].

With regard to executive functions (EF), which are considered a central source of the disability associated with ADHD [16-19], previous research has yielded more similarities than differences between girls and boys with ADHD [11,20]. Despite the centrality of EF deficits in ADHD, a neuropsychological profile distinct to females with ADHD when compared with male counterparts has yet to be identified. In addition, neuropsychological tests have shown to be weakly related to the severity of ADHD symptoms [21], and recent studies suggest that many subjects with ADHD perform normally on EF tests [22,23]. Where traditional neuropsychological EF tests seem to capture “best estimates” in an ideal setting [24], the Behavior Rating Inventory of Executive Function (BRIEF) was designed to assess EF performance in ecologically valid situations [25]. This instrument has shown consistent, but modest correlations with neuropsychological EF tests [26-28]. The BRIEF has proven to be a valuable additional assessment tool for the identification of ADHD in school-aged children [28,29], demonstrating better sensitivity than neuropsychological EF tests [28]. To the best of our knowledge, no studies have investigated potential gender effects on BRIEF in children and adolescents with ADHD. However, Huizinga and colleagues [30] reported elevated levels of executive problems assessed with BRIEF in normal developing boys compared to normal developing girls (age range 5–18 years). These findings are comparable to the data presented in the original version of the BRIEF, showing superior performance in girls compared to boys in general [25].

In sum, the gender gap in clinical populations of subjects with ADHD continues to hamper the correct diagnosis and treatment of females with ADHD. To our knowledge, the current study is the first to include both self-report and parent ratings of co-existing symptoms in addition to laboratory testing and inventory based scales assessing EF in medication naïve boys and girls with ADHD to examine potential gender sensitive ADHD profiles.

Our first aim was to assess whether there were significant gender x diagnosis interactions in co-existing symptom severity and EF impairment. Using conventional ANOVAs we hypothesised that boys and girls with ADHD would show greater impairment in all three measurement domains (co-existing symptoms, neuropsychological EF tests, BRIEF) relative to HC. However, we expected to find few significant gender x diagnosis interactions in the three measurement domains. Our second aim was to delineate specific symptom ratings and measures of EF that were most important in distinguishing ADHD from HC in the same gender. We used random forest classification with cross-validation, where the identification of subtle differences across diagnosis and gender in moderately sized samples is possible. It was hypothesised that co-existing internalizing symptoms would better distinguish subjects with ADHD from HC in females than in males. Co-existing externalizing symptoms would better distinguish subjects with ADHD from HC in males than in females. Second, neuropsychological test results were expected to distinguish subjects with ADHD from HC equally well in males and females. Finally, parent ratings of EF were hypothesised to better distinguish subjects with ADHD from HC in males than in females.

## Methods

### Procedure and participants

Demographic characteristics are presented in Table 1. Forty three males with ADHD (*M* = 11.2 years), 37 females with ADHD (*M* = 11.9 years), 32 healthy control (HC) males (*M* = 11.4 years) and 18 HC females (*M* = 11.9 years) between 8 and 17 years participated in the study. The ADHD participants were recruited as consecutive referrals from seven outpatient Child and Adolescent Mental Health Centres in Innlandet Hospital Trust (IHT) for assessment of ADHD. All participants underwent a comprehensive assessment according to common clinical practice. Semi-structured clinical interviews (Kiddie-Schedule for Affective Disorders and Schizophrenia - K-SADS) [31] were conducted separately for children/adolescents and parents to assess psychopathology. The interviewers were experienced clinicians, and were trained to high levels of interrater reliability for the assessment of diagnosis. The diagnostic evaluation with K-SADS was supplemented with information from the ADHD Rating Scale IV (ARS-IV) [32], and the Child Behavior Checklist/6-18 [33], which covers the DSM-IV symptoms for ADHD. Teacher reports describing school functioning, both academic and socially, which is mandatory on referral, were incorporated into the diagnostic evaluation. Diagnoses were considered positive if, based on a comprehensive evaluation of K-SADS, teacher information and rating scales, DSM-IV [1] criteria were met.

**Table 1:** Demographic characteristics: means and standard deviations within the four groups

	**ADHD**		**Healthy controls**				
**Variable**	**Boys**^***1 ***^**(*n *** **= 43)**	**Girls**^***2 ***^**(*n *** **= 37)**	**Boys**^***3 ***^**(*n *** **= 32)**	**Girls**^***4 ***^**(*n *** **= 18)**	**Group comparison**		Bonferroni^*^
					***F***	***P***	
Age (months)	139.2 (23.2)	149.4 (25.1)	141.5 (22.6)	148.5 (27.3)	*F* (3,126) = 1.50	NS	
Mother’s education (yrs)	12.6 (2.3)	12.9 (1.9)	14.6 (2.5)	14.6 (2.1)	*F* (3,126) = 6.80	< .001	1 < 3,4; 2 < 3
FSIQ (WASI)^a)^	94.3 (13.2)	96.4 (15.5)	101.9 (12.7)	107.1 (13.1)	*F* (3,126) = 4.60	= .004	1,2 < 4
Inattention^b)^	16.6 (5.8)	15.0 (5.6)	1.8 (2.1)	1.4 (1.6)	*F*(3,125) = 95.50	< .001	1,2 < 3,4
Hyperactivity/ Impulsivity^c)^	10.2 (6.5)	10.1 (6.7)	1.1 (1.3)	0.8 (1.4)	*F* (3,125) = 30.86	< .001	1,2 < 3,4
CGAS^d)^	56.0 (8.4)	59.3 (9.3)			*t* (78) = 2.82	NS	

^a)^Full Scale IQ.

^b)^ADHD rating scale – IV.

^c)^ADHD rating scale – IV.

^d)^Children Global Assessment Scale.

^*^^1^ = ADHD Boys, ^2^ = ADHD Girls, ^3^ = HC Boys, ^4^ = HC Girls.

Based on diagnostic evaluation with K-SADS, co-existing diagnoses within the group of males with ADHD included depression (4.7%), anxiety (4.7%), conduct disorder (4.7%), and oppositional defiant disorder (11.6%). Co-existing diagnoses within the females with ADHD included anxiety (8.1%), and oppositional defiant disorder (10.8%). Despite a low prevalence of co-existing diagnoses, parent and self-report scales indicated elevated levels of externalizing and internalizing symptoms in both males and females with ADHD when compared with normal developing counterparts. Exclusion criteria for all participants included prematurity (< 36 weeks), IQ below 70, a history of stimulant treatment or any disease affecting the central nervous system. None of the participants used any type of psychopharmacological medication. One boy with ADHD was excluded due to estimated IQ below 70. None were excluded due to history of stimulant treatment or any disease affecting the central nervous system.

All participants in the HC groups were screened for mental disorders with the K-SADS in separate interviews for children/adolescents and parents. The HC were recruited from local schools and were given a small compensation for participating. The HC could not have been treated for a mental disorder, have a psychiatric diagnosis, have had a head injury (with loss of consciousness) or known dyslexia. The four groups (ADHD/females, ADHD/males, HC/females, HC/males) did not differ significantly with regard to age and gender distribution. The Wechsler Abbreviated Scale of Intelligence (WASI) [34] was administered to estimate IQ in all participants. The groups differed significantly with regard to IQ, *F* (3,126) = 4.60, *p* = .004, Eta^2^ = .099, and Bonferroni post-hoc analysis showed that both ADHD gender groups scored below the females in the HC group. On average, mothers of children in the HC group had 1.7 years more education than mothers of children with ADHD, *F* (3,126) = 6.80, *p* < 0.001. All parents/caregivers and participants above 12 years gave written informed consent in accordance with the Research Ethics Committee in Eastern Norway. All children under the age of 12 years provided oral consent to participate. The study was approved by the Regional Committee for Medical Research Ethics in Eastern Norway (REK-Øst), and by the Privacy protection ombudsman for research at Innlandet Hospital Trust. It was conducted in accordance with the Helsinki Declaration of the World Medical Association Assembly.

### Measures

#### Measures of symptomatology

The Child Behavior Checklist/6-18 (CBCL) [33] is a widely used scale containing 7 competence items and 113 specific problem items, each of which is rated on a 0–2 metric. The 120 items assess adaptive behavior as well as eight narrow band factors (Anxious/Depressed, Withdrawn/Depressed, Somatic Complaints, Social Problems, Thought Problems, Attention Problems, Rule-Breaking Behavior and Aggressive Behavior) and two broadband factors (Externalizing and Internalizing symptoms) of co-existing symptoms. The 2001 revision also includes seven DSM-oriented scales consistent with DSM diagnostic categories (Affective Problems, Anxiety Problems, Somatic Problems, ADHD, Oppositional Defiant Problems and Conduct Problems). On the parent-report CBCL, we used seven of the narrow band factors (excluding Attention Problems) and five of the DSM-oriented scales (excluding ADHD) to assess co-existing symptoms. Elevated T-scores indicate a higher degree of co-existing internalizing and externalizing symptoms. Cross-cultural studies have demonstrated good discriminant validity with mean factor loadings across societies at .62 [35]. Acceptable reliability and validity of the Norwegian version of the CBCL are reported by Nøvik [36,37].

The Revised Children’s Manifest Anxiety Scale, second edition (RCMAS-2) [38] is a 49-item self-report instrument designed to measure anxiety symptoms in children 6 to 19 years of age. Children respond either “Yes” or “No” to all 49-items. The instrument reveals three anxiety factors: Physiological Anxiety, Worry and Social Anxiety. The three anxiety factors are summed yielding a Total Anxiety score. Elevated raw-scores indicate a higher degree of anxiety symptoms. The RCMAS Total Anxiety Scale has been found to have satisfactory psychometric properties with high test–retest reliability [39,40] and consistent construct validity [41-44]. Satisfactory psychometric properties have been replicated among other cultures as well [39,45-47].

The State-Trait Anxiety Inventory for Children (STAIC) [48] includes two 20-item self-report scales that measure both enduring tendencies (Trait) and situational variations (State) in levels of perceived anxiety. Children respond on a three-point scale indicating varying degree of worry, feelings of tension, and/or nervousness. Elevated raw-scores indicate a higher degree of situational and temporal anxiety. In a quantitative review by Seligman and colleagues [49], the authors argue that the STAIC possess satisfactory psychometric properties.

The Short Mood and Feelings Questionnaire (SMFQ) [50] is a 13-item self-report instrument designed to measure depressive symptoms in children 8 to 18 years of age. The SMFQ is derived from the original 30-item Mood and Feelings Questionnaire (MFQ) [51] where children respond on a three-point scale (“not true”, “sometimes true” and “true”). A net score was generated based on the 13 items with elevated raw-scores indicating a higher degree of depression symptoms. The SMFQ have demonstrated high internal consistency (Crohnbach’s alpha = .90) [52], and test-retest stability in children for a two-week period yielded an intra class correlation of .66 [51]. Angold and colleagues [50] found SMFQ to correlate strongly with Children’s Depression Inventory (CDI) [53] and Diagnostic Interview Schedule for Children (DISC-C) depression scores [51] (*r* = .67 and .51, respectively).

#### Neuropsychological EF tests

### The letter-number sequencing test

The Letter-Number Sequencing Test (LN) [54] was used as a measure of working memory. The test consists of ten items. Each item contains three trials with the same number of digits and letters. The test administrator reads aloud each trial and asks the child to recall the numbers in ascending order and the letters in alphabetical order. In the present study, total correct recalled trials were examined. Lower scaled scores indicated difficulties with the task.

#### The colour - word interference test, condition 3

The Colour - Word Interference Test, Condition 3 (CW 3) [55,56] was used as a measure of inhibition. The examinee needs to inhibit an overlearned verbal response when naming the dissonant ink colours in which the words are printed. For the present study, completion time in seconds was examined. Lower scaled scores indicated difficulties with the task.

#### The colour - word interference test, condition 4

The Colour - Word Interference Test, Condition 4 (CW 4) [56] was used as a measure of cognitive flexibility. The examinee is asked to switch back and forth between naming the dissonant ink colours and reading the words. For the present study, completion time in seconds was examined. Lower scaled scores indicated difficulties with the task.

#### The trail making test, condition 4

The Trail Making Test, condition 4 (TMT 4) [56] was used as a measure of cognitive flexibility. The examinee is asked to draw a line interchangeably between numbers and letters in the right order. For the present study, time to complete task was examined. Lower scaled scores indicated difficulties with the task.

#### The design fluency test, condition 3

The Design Fluency Test, condition 3 (DF) [56] was used as a measure of cognitive flexibility. The examinee is asked to draw as many different designs as possible using four straight lines connecting five filled and empty dots interchangeably. The examinee is given 60 seconds for the task. For the present study, total correct responses were examined. Lower scaled scores indicated difficulties with the task.

#### The tower test

The Tower Test [56] was used as a measure of planning. In this task the examinee is asked to construct several target towers by moving five disks, varying in size, across three pegs in the fewest number of moves possible. While doing this, the examinee is allowed to move only one disk at a time, and not to place a larger disk over a smaller disk. In the present study total achievement score was examined. Lower scaled scores indicated difficulties with the task.

#### The letter fluency test

The Letter Fluency Test (LF) [56] was used as a measure of verbal fluency. This task includes three 60-seconds trials, where participants were asked to generate words fluently in an effortful, phonemic format with the letters F, A, and S. For the present study, total correct responses were examined. Lower scaled scores indicated difficulties with the task.

#### Inventory based information of EF

The BRIEF for children and adolescents aged 5 to 18 includes a parent form and a teacher form [25]. In the current study, the Norwegian parent rating version was used. The BRIEF is composed of eight clinical scales (Inhibition, Shift, Emotional Control, Initiate, Working Memory, Plan/Organize, Organization of Materials and Monitor). Fallmyr & Egeland [57] reported high internal consistency (Chronbachs α = .76-.92) on the Norwegian parent rating version of the BRIEF. These values are at the same level as Chronbachs α reported in the BRIEF manual (.80-.98) [25]. Elevated BRIEF T-scores indicate a higher degree of impairment.

### Data analyses

Data analyses were conducted using the statistical package SPSS for Windows, version 15.0 (SPSS, Inc., Chicago, IL). Demographic characteristics were investigated using the Chi-square test for independence (nominal variables) and analysis of variance (ANOVA) (continuous variables) followed up by Bonferroni post-hoc tests for group comparisons when adequate. ANOVAs were carried out to investigate gender x diagnosis interactions in the three measurement domains (co-existing symptoms, neuropsychological EF tests, BRIEF).

#### Random forest classification

In addition to tests of significance we also used an algorithmic modelling/data mining technique to explore gender differences in co-existing symptoms and EF ratings and measures. Classical statistical techniques are designed to test and reject the hypothesis that observed differences between groups have occurred by chance. Algorithmic modelling techniques have been developed to address a somewhat different question. Briefly, these techniques can identify from a sample of potential predictor variables the most important subset for categorizing subjects or predicting outcomes [58]. Hence, we used this approach to delineate within each gender the subset of symptom ratings, EF measures and EF ratings that appear to be most important in discriminating children with ADHD from HC. Specifically, we used random forest classification and cross-validation (R packages *randomForest 4.5*-*34* and *caret 5.02*-*011*) [59] to identify and rank order different symptom ratings and EF measures for their degree of importance in differentiating ADHD from HC within each gender. Although importance and statistical significance often go hand-in-hand, the two are not necessarily the same. The approach has many advantages. In particular, it can provide meaningful results with smaller sample sizes than stochastic models. Further they are less susceptible to overfitting and multicollinearity, provide more accurate predictions, and do not make the unlikely assumption that the multivariate data being analyzed are multivariate normal.

Briefly, this is a form of “ensemble learning” in which a large number of unpruned decision trees are generated and their results aggregated [60]. The random part comes in as each tree is constructed using a different bootstrap sample of the data, and each node is split using the best among a subset of predictors randomly chosen at that node. As Liaw and Wiener indicate [61] this strategy performs very well compared to many other classifiers, including discriminant analysis, logistic regression, support vector machines and neural networks [60]. It is primarily used in data mining and in genomic analysis, such as microarray studies.

Each decision tree was generated using results from 75% of the participants and then tested on the remaining 25% (validation set). This process was performed 5000 times on different random splits of the data to provide a cross-validated estimate of the predictive discriminant ability of the measures (accuracy, kappa) that would likely generalize to new cases [62]. The importance of each variable in the cluster was assessed by calculating the decrease in predictive accuracy following the sequential permutation (effective randomization and elimination) of each variable in the cluster on the validation set. The most important variables were the ones whose effective elimination from the forest produced the greatest degradation in accuracy.

## Results

The first statistical approach tested the hypothesis that there were significant gender x diagnosis interactions across the array of dependent variables (DVs), viewing each of the DVs in isolation. In the second approach we evaluated the ability of ratings or measures in the: (1) symptom (CBCL, RCMAS-2, STAIC, SMFQ), (2) EF test performance, and (3) EF rating (BRIEF) clusters to predict whether participants met criteria for ADHD, and if the most important predictor variables in each cluster were the same for males and females.

As seen in Table 2 there were marked group differences between participants with ADHD and HC in symptom ratings. In general, there was a roughly parallel increase in symptom ratings with diagnosis across gender, and the only significant diagnosis x gender interaction was observed in ratings of physiological anxiety on the RCMAS-2. There were significant main effects of diagnosis on several of the EF measures including: working memory (LN), inhibition (CW3), and cognitive flexibility (CW4, TMT4, DF). However, there were no significant gender x diagnosis interactions on these measures (Table 3). Interestingly, there were also robust group differences between parent ratings of children with ADHD and of HC on the BRIEF (Table 4). However, on none of the BRIEF ratings were there significant diagnosis by gender interactions.

**Table 2:** Group differences and interaction effects on symptom ratings (ANOVA): means and standard deviations

	**Group**				**Main and interaction effects**					
	**ADHD**		**Healthy controls**							
	**Boys (n = 43)**	**Girls (n = 37)**	**Boys (n = 32)**	**Girls (n = 18)**	**Group**		**Gender**		**Group X Gender**	
					***F***	***p***	***F***	***p***	***F***	***p***
**CBCL**^*a*)^										
Anxious/depressed	58.3 (8.2)	61.1 (10.6)	51.5 (4.0)	51.4 (2.7)	31.11	<.001	1.52	NS	0.98	NS
Withdrawn/depressed	59.4 (8.2)	58.7 (7.2)	51.5 (2.5)	51.2 (2.6)	45.29	<.001	0.26	NS	0.04	NS
Somatic complaints	59.6 (8.9)	59.7 (8.6)	53.0 (4.7)	51.4 (2.5)	29.25	<.001	0.30	NS	0.35	NS
Social problems	60.4 (9.2)	60.0 (7.4)	50.5 (1.5)	50.3 (0.5)	65.08	<.001	0.02	NS	0.00	NS
Thought problems	58.1 (8.8)	57.2 (7.7)	51.1 (2.1)	50.6 (1.5)	31.40	<.001	0.36	NS	0.02	NS
Rule-breaking	60.3 (8.6)	58.6 (8.0)	50.9 (2.6)	50.8 (2.5)	52.06	<.001	1.59	NS	0.52	NS
Aggressive behavior	61.8 (11.7)	61.5 (9.6)	50.9 (1.8)	50.7 (2.1)	47.81	<.001	0.03	NS	0.00	NS
Affective problems	61.8 (8.7)	63.2 (8.8)	51.4 (3.6)	50.9 (1.7)	72.89	<.001	0.27	NS	0.47	NS
Anxiety problems	57.0 (7.8)	60.7 (8.4)	51.2 (3.4)	51.2 (3.3)	35.13	<.001	4.08	.045	2.21	NS
Somatic problems	60.0 (8.6)	60.1 (10.3)	53.4 (5.0)	51.6 (2.9)	25.96	<.001	0.17	NS	0.43	NS
Oppositional problems	59.7 (8.4)	59.8 (8.3)	51.3 (2.2)	51.5 (2.7)	45.75	<.001	0.00	NS	0.00	NS
Conduct problems	62.6 (9.8)	60.1 (8.3)	51.2 (2.6)	50.9 (2.4)	61.60	<.001	2.18	NS	0.80	NS
**SMFQ**^*b*)^	6.3 (4.9)	7.7 (4.9)	2.4 (2.8)	1.7 (1.6)	38.83	<.001	0.70	NS	1.91	NS
**RCMAS-2**^*c*)^										
Physiological anxiety	5.1 (2.2)	6.5 (2.3)	2.6 (2.2)	1.4 (1.6)	82.35	<.001	1.61	NS	10.46	.002
Worry	4.2 (4.0)	6.3 (3.8)	2.1 (1.8)	2.0 (1.9)	25.06	<.001	4.83	.030	3.40	NS
Social anxiety	3.6 (3.2)	5.4 (2.7)	1.3 (1.6)	1.4 (1.7)	39.42	<.001	5.94	.016	2.95	NS
**STAIC**^*d*)^										
State	29.2 (4.1)	30.1 (5.0)	27.3 (4.0)	27.4 (3.4)	8.21	.005	0.23	NS	0.16	NS
Trait	30.5 (8.5)	34.4 (7.9)	27.7 (5.2)	26.5 (5.9)	13.42	<.001	2.39	NS	3.67	NS

^*a*)^*The Child Behavior Checklist*/*6*-*18* (*df* = 1,124). ^*b*)^*The Short Mood and Feelings Questionnaire* (*df* = 1,123). ^*c*)^*The Revised Children*’*s Manifest Anxiety Scale*, *second edition* (*df* = 1,124). ^*d*)^*The State*-*Trait Anxiety Inventory for Children*: State (*df* = 1,121), Trait (*df* = 1,122).

Note. Higher scores denote greater pathology.

**Table 3:** Group differences and interaction effects on executive function measures (ANOVA): means and standard deviations

	**Group**				**Main and interaction effects**					
	**ADHD**		**Healthy controls**							
	**Boys (n = 43)**	**Girls (n = 37)**	**Boys (n = 32)**	**Girls (n = 18)**	**Group**		**Gender**		**Group X Gender**	
					***F***	***p***	***F***	***p***	***F***	***p***
LN^*a*)^	8.2 (2.9)	8.6 (2.6)	10.6 (1.5)	10.9 (1.7)	29.39	<.001	0.85	NS	0.01	NS
TMT4^*b*)^	7.1 (3.2)	7.8 (3.3)	9.3 (2.9)	10.2 (2.5)	17.14	<.001	2.24	NS	0.00	NS
CW3^*c*)^	8.4 (3.3)	8.3 (3.4)	10.0 (2.4)	11.5 (1.7)	17.40	<.001	1.11	NS	2.17	NS
CW4^*d*)^	7.9 (2.9)	7.1 (3.5)	10.0 (2.6)	10.9 (1.6)	28.46	<.001	0.07	NS	2.76	NS
Tower^*e*)^	9.9 (2.2)	10.6 (2.3)	11.0 (2.1)	10.3 (1.7)	1.93	NS	0.13	NS	3.26	NS
DF^*f*)^	8.2 (2.5)	9.8 (3.8)	11.8 (2.9)	12.3 (2.4)	31.51	<.001	4.82	.030	0.83	NS
LF^*g*)^	8.2 (0.5)	9.1 (0.3)	11.3 (0.5)	11.8 (0.9)	26.21	<.001	1.53	NS	0.06	NS

^*a*)^*The Letter*-*Number Sequencing Test* (*df* = 1,123). ^*b*)^*The Trail Making Test*, *condition 4* (*df* = 1,123). ^*c*)^*The Colour*-*Word Interference Test*, *condition 3* (*df* = 1,125). ^*d*)^*The Colour*-*Word Interference Test*, *condition 4* (*df* = 1,125). ^*e*)^*The Tower Test* (*df* = 1,124). ^*f*)^*The Design Fluency Test*, *condition 3* (*df* = 1,116). ^*g*)^*The Letter Fluency Test* (*df* = 1,123).

**Table 4:** Group differences and interaction effects on executive function ratings (ANOVA): means and standard deviations

	**Group**				**Main and interaction effects**					
	**ADHD**		**Healthy controls**							
	**Boys (n = 43)**	**Girls (n = 37)**	**Boys (n = 32)**	**Girls (n = 18)**	**Group**		**Gender**		**Group X Gender**	
					***F*** *	***p***	***F*** *	***p***	***F*** *	***p***
Inhibit	59.7 (15.9)	62.5 (12.9)	42.3 (3.5)	42.6 (3.3)	75.17	<.001	0.60	NS	0.31	NS
Shift	57.5 (13.7)	57.3 (11.3)	40.9 (5.8)	40.6 (3.1)	77.65	<.001	0.09	NS	0.00	NS
Emotional control	59.0 (14.1)	61.3 (13.7)	40.7 (3.7)	41.4 (5.3)	88.14	<.001	0.32	NS	0.12	NS
Initiate	59.2 (11.5)	61.8 (12.1)	41.0 (7.2)	40.1 (5.8)	112.18	<.001	0.70	NS	0.96	NS
Working memory	69.3 (9.9)	70.5 (10.1)	41.6 (4.9)	42.8 (4.1)	333.64	<.001	0.56	NS	0.00	NS
Plan/Organize	64.6 (9.8)	67.0 (10.3)	41.4 (5.3)	41.3 (4.1)	250.37	<.001	0.66	NS	0.60	NS
Organization of materials	56.7 (11.4)	58.3 (10.8)	41.3 (7.8)	42.1 (7.4)	75.41	<.001	0.63	NS	0.06	NS
Monitor	60.3 (11.9)	63.8 (12.3)	37.9 (6.1)	39.7 (4.5)	160.68	<.001	1.91	NS	0.17	NS

*df = 1,125.

Note: Elevated BRIEF T-scores indicate a higher degree of impairment, with T-scores of 65 and above considered to represent clinically significant areas of concern.

**Figure 1:**
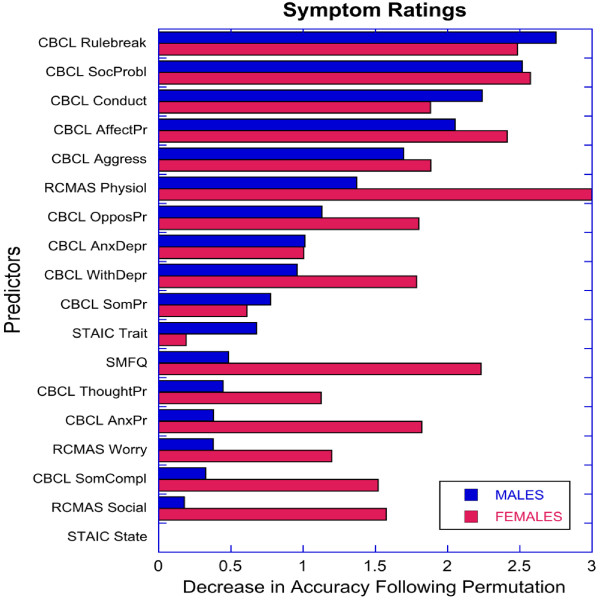
Relative importance of symptoms ratings in predicting ADHD status by gender with random forest classification.

Random forest classification investigating the relationship between ADHD status and non-ADHD symptom cluster had a predictive (i.e., cross-validated) diagnostic accuracy of 0.860 ± 0.058 (mean ± SD) in males and 0.932 ± 0.055 in females. Kappa coefficients were 0.715 ± 0.115 and 0.844 ± 0.133 for males and females, respectively. This indicates that random forest classification using non-ADHD symptom ratings was substantially better at discriminating female subjects with ADHD from female HC than they were in discriminating males with ADHD from male HC (accuracy: z = 5.8, *p* < 10^-8^; kappa: z = 4.7, *p* < 10^-5^; two-sample Z-test). As seen in Figure 1, the rank order pattern of variable importance for discriminating ADHD from HC was similar in males and females (r_s_ = 0.676, *p* < 0.003). CBCL ratings of social problems and affective problems were key distinguishing variables in both genders. However, rule breaking was the most important distinguishing variable in males, while physiological anxiety symptoms on the RCMAS-2 was the most important distinguishing variable in females.

**Figure 2:**
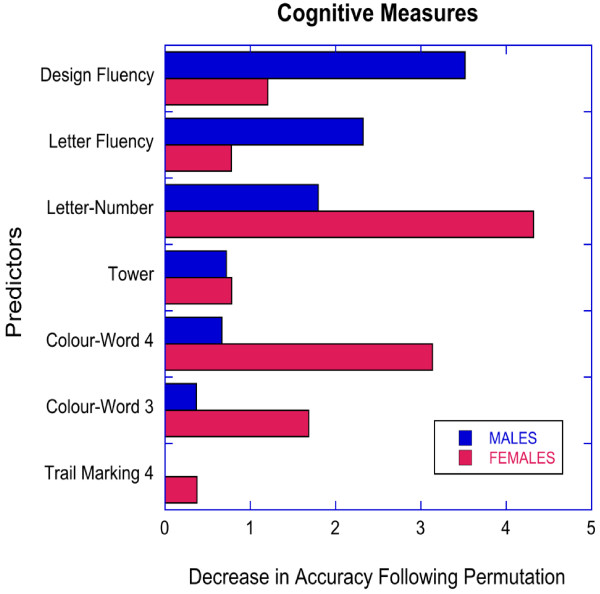
Relative importance of executive function measures in predicting ADHD status by gender with random forest classification.

Random forest classification investigating the relationship between ADHD status and EF tests assessing: working memory, inhibition, cognitive flexibility, planning, and verbal fluency had only a modest ability to distinguish participants with ADHD from HC (males: accuracy = 0.734 ± 0.078, kappa = 0.466 ± 0.152; females: accuracy = 0.785 ± 0.078, kappa = 0.507 ± 0.175). There was a significant gender difference in the predictive ability of random forests based on measures of accuracy but not kappa (accuracy: z = 3.22, *p* < 0.001; kappa: z = 1.22, *p* < 0.12). As seen in Figure 2, the rank order patterning of variable importance on these measures were not significantly correlated between genders (r_s_ = 0.143, *p* > 0.7). The most important distinguishing variables in males were performance on tests assessing cognitive flexibility (DF) and verbal fluency (LF). The most important distinguishing variables in females were performance on tests assessing working memory (LN), inhibition (CW 3), and cognitive flexibility (CW 4).

**Figure 3:**
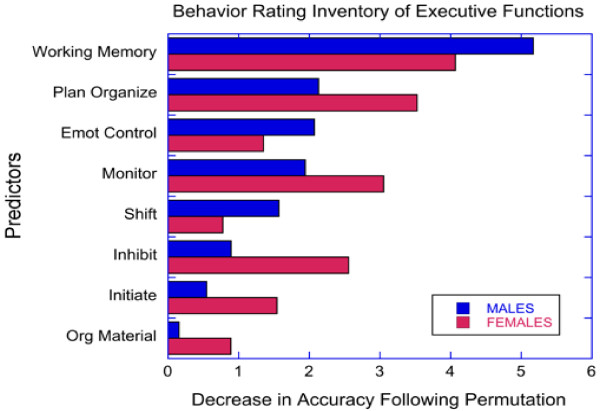
Relative importance of executive functions ratings (BRIEF) in predicting ADHD status by gender with random forest classification.

Random forest classification investigating the relationship between ADHD status and BRIEF showed that parent ratings of executive skills were able to discriminate males with ADHD from male HC with high accuracy (0.960 ± 0.036, kappa 0.916 ± 0.076). Random forest classification using BRIEF items was not quite as accurate in discriminating females with ADHD from female HC (accuracy = 0.923 ± 0.051, z = 4.29, *p* < 10^-5^ versus males; kappa = 0.818 ± 0.123, z = 4.85, *p* < 10^-6^). As seen in Figure 3, BRIEF working memory was the most important distinguishing variable in both genders. However, the rank ordering of importance of the eight BRIEF variables between genders correlated to only a marginal degree (r_s_ = 0.667, *p* = 0.07).

## Discussion

As expected, results on non-ADHD symptom ratings, EF ratings and EF measures differed substantially between ADHD subjects and HC. Boys and girls with ADHD showed in general greater impairment in all three measurement domains relative to HC. There was little evidence for diagnosis x gender differences in mean ratings or measures, with more self-reported physiological anxiety in females with ADHD relative to the other groups, as the only significant diagnosis x gender finding. Elevated levels of co-existing internalizing symptoms in females with ADHD relative male counterparts is in accordance with the study by Rucklidge & Tannock [14]. However, few diagnosis x gender differences in general is consistent with previous reports from population-based studies indicating that the disorder is expressed similarly in boys and girls [8,63]. On the other hand, random forest classification with cross-validation provided evidence for meaningful gender differences when investigating the relationship between ADHD status and the three measurement clusters, and in the relative importance of specific items.

First, random forest regression with cross-validation of the symptom cluster indicated that non-ADHD symptoms appeared to be better at categorizing participants as HC or ADHD in females than in males. Our results corroborate previous findings by Rucklidge & Tannock [14] reporting that parent and teachers reported more difficulties with oppositional behaviors, conduct problems, social difficulties, anxiety, and depression in females with ADHD compared to male counterparts. Together these findings provide evidence that co-existing psychological impairment may be even more reliably evident in females with ADHD compared to HC females than in males with ADHD relative to HC males.

Consistent with our hypothesis random forest regression with cross-validation indicated that the most important non-ADHD symptom for categorizing females as ADHD or HC was increased self-reported physiological anxiety (internalizing symptoms), whereas the most important symptom in males was parent rated rule breaking (externalizing symptoms). Elevated ratings of somatic complaints in girls with ADHD have previously been reported in population based studies [64], and supports a hypothesis of somatic complaints as markers for anxiety proneness in females with ADHD [65,66]. Elevated levels of externalizing symptoms in males with ADHD have been documented in several research reviews [3,5,9].

Third, random forest regression with cross-validation showed that neuropsychological measures of EF had only a modest ability to categorize participants as ADHD or HC. Differences in accuracy when categorizing participants as HC or ADHD in females versus males were slight (79% versus 73% accuracy), and were consistent with our hypothesis of no major difference in discriminatory power between genders. These results corroborate previous findings reporting moderate validity of EF tests for classifying children with ADHD [18,23]. Interestingly, there were differences between genders in the specific EF measures that appeared to be the most important distinguishing variables. Cognitive flexibility and verbal fluency were the most important distinguishing variables in males, whereas working memory and inhibition were the most important distinguishing variables in females. A few previous studies have reported sex differences in EF in children with ADHD [67,68] though most studies report similar EF profiles [11,20]. In sum, EF tests show limited sensitivity and specificity for classifying boys and girls with ADHD.

Fourth, random forest regression with cross-validation indicated that parental ratings of EF were relatively robust distinguishing variables of ADHD status in this sample. In line with our hypothesis, BRIEF scales was significantly better at discriminating males with ADHD from HC males than they were in discriminating females with ADHD from female HC. While there were differences in the relative importance of the different EF ratings, working memory appeared to be the most important distinguishing variable in both boys and girls. The sensitivity of working memory ratings in distinguishing boys and girls with ADHD from HC has been previously documented by Isquith & Gioia [69] and McCandless & O’Laughlin [29]. Our results indicating better discriminatory power in males than in females with the BRIEF, suggests that ADHD in males may be more reliably associated with impairments in EF than in females. This higher risk of behavioral problems in males with ADHD symptoms may be one reason for the referral of more boys than girls for clinical evaluation of ADHD, as previously suggested by Gaub and Carlson [3].

In our study, the participants’ symptoms were assessed with both self-report and parent rating scales. Previous research has shown that clinical samples of children often report more symptoms about themselves than parents do with regard to anxiety and depression [70-74]. In contrast, parents are found to report more conduct disorders [72] or behavioral symptoms than their children [75-77]. Our results with self-reported internalizing symptoms (physiological anxiety) in females with ADHD and parent rated functional impairment in males with ADHD reflect previous reports regarding a self-report/parent rating discrepancy. As internalizing symptoms are less readily observed, parental reports of anxiety and depression are less sensitive than parental reports of externalizing behaviors [77]. It has been speculated that high levels of anxiety and depression in females with ADHD indicate that females are more negatively affected than their male counterparts [78]. The inclusion of self-report scales in clinical practice may thus increase awareness of internalizing problems particularly salient in females with ADHD, and intervention should target co-existing anxiety and depression when indicated. Where parent ratings seem to be informative regarding behavioral problems in boys with ADHD, self-report scales may be more informative regarding internalizing problems in girls with this disorder. Thus, clinical intervention should be sensitive to different expression in co-existing symptoms for boys and girls with ADHD, in addition to conventional treatment of ADHD symptoms.

Strengths of the present study are inclusion of subjects not medicated with psychopharmaca and no history of stimulant treatment. Additional strengths are the use of both self-report and parent ratings when assessing co-existing symptoms, and laboratory tests and inventory-based scales assessing EF. Further, random forest classification [60] is a relatively novel method of determining variable importance, with the advantages of very high classification accuracy and no restrictions regarding the distribution and scaling properties of the data [79]. These properties make random forest regressions well-suited for the classification of large sets of data. It is interesting in this context that predictive modelling with random forests identified gender differences in the accuracy of cluster based categorizations and in the importance of specific measures, whereas conventional statistical analyses showed only one instance of a significant diagnosis by gender interactions. This is basically a consequence of limited power of the ANOVA test to detect small or subtle interactions. Significant interactive effects are typically detected when the opposite response pattern is seen across gender, or when a large effect is present in one gender and a small effect is present in the other. In order to detect a subtle interactive effect (*f* = 0.1) with power of 0.8 would have required a much larger sample size (n = 787).

We used random forests with cross-validation to simultaneously assess clusters of variables to delineate predictive accuracy and to identify the most important distinguishing variables. This approach has been shown to be effective in identifying the most important predictors in large sets of variables with fewer participants then can even be considered using conventional statistical techniques [60]. For instance, this approach can be used in microarray studies to accurately identify the most important subset of polymorphisms even when the number of variables greatly exceeds the number of participants. In short, this approach makes it possible to identify subtle differences across diagnosis and gender in symptom measures and ratings in moderately sized samples.

The present findings need to be interpreted in the context of some methodological limitations. Participants were recruited from a sample of referred subjects, and consequently are not necessarily representative of children and adolescents with ADHD in the general population. However, we believe the participants are fairly representative of males and females referred for the evaluation of ADHD related symptoms. Previous findings have reported that females with ADHD present more commonly with the inattentive subtype than do boys [10]. These subtype effects may potentially have an impact on gender dependent symptom profiles observed in our study. However, despite an overload of males in the ADHD-C group (23 males, 13 females), subtype distribution did not differ significantly by gender in our sample, and the level of inattention and hyperactivity/impulsivity symptoms (ARS-IV) [32] was equal between males and females with ADHD. Albeit previous studies have reported gender differences in hyperactive/impulsive and inattentive symptoms [3,8,9], findings by Lambek and colleagues [80] indicated that these gender dependent subtype effects may be more evident in non-referred than in referred samples of boys and girls with ADHD.

## Conclusions

Overall, females with ADHD could be more accurately distinguished from HC by the presence of co-existing symptoms, particularly physiological components of anxiety. On the other hand, parental reports of EF impairments were substantially better at distinguishing subjects with ADHD from HC in males. Given the almost universal phenomenon of “co-morbidity” in ADHD, our findings emphasize the combined value of self-report and parent rating scales for the identification of comorbid symptoms in boys and girls already diagnosed with ADHD.

## Competing interests

The authors declare no conflict of interest with respect to authorship or publication of this article.

Parts of this paper were presented as a poster at the Eunethydis 2nd International ADHD Conference in Barcelona 23–25 May 2012.

## Authors’ contributions

EWS managed the literature searches, undertook the preliminary statistical analyses, interpreted the data, and wrote the first draft of the current manuscript. MHT undertook the statistical analyses, helped interpret the data, and revised the current manuscript critically. PNA helped collect the data, and revised the current manuscript critically. KTH helped collect the data, and revised the manuscript critically. MØ wrote the protocol, managed the literature searches, helped interpret the data and revised the current manuscript critically. All consented to their names on the final manuscript. All authors read and approved the final manuscript.

## Pre-publication history

The pre-publication history for this paper can be accessed here:

http://www.biomedcentral.com/1471-244X/13/298/prepub
